# Association between neutrophil-to-lymphocyte ratio and diabetic kidney disease in type 2 diabetes mellitus patients: a cross-sectional study

**DOI:** 10.3389/fendo.2023.1285509

**Published:** 2024-01-04

**Authors:** Xiaowan Li, Lanyu Wang, Min Liu, Hongyi Zhou, Hongyang Xu

**Affiliations:** ^1^ Department of Critical Care Medicine, The Affiliated Wuxi People’s Hospital of Nanjing Medical University, Wuxi People’s Hospital, Wuxi Medical Center, Nanjing Medical University, Wuxi, China; ^2^ Department of Urology, The Affiliated Wuxi People’s Hospital of Nanjing Medical University, Wuxi People’s Hospital, Wuxi Medical Center, Nanjing Medical University, Wuxi, China

**Keywords:** neutrophil-to-lymphocyte ratio, type 2 diabetes mellitus, diabetic kidney disease, population-based study, NHANES

## Abstract

**Aims:**

This investigation examined the possibility of a relationship between neutrophil-to-lymphocyte ratio (NLR) and diabetic kidney disease (DKD) in type 2 diabetes mellitus (T2DM) patients.

**Methods:**

Adults with T2DM who were included in the National Health and Nutrition Examination Survey (NHANES) between 1999 and 2020 were the subjects of the current cross-sectional investigation. Low estimated glomerular filtration rate (eGFR) (< 60 mL/min/1.73 m^2^) or albuminuria (urinary albumin-to-creatinine ratio (ACR) ≥ 30 mg/g) in T2DM patients were the diagnostic criteria for DKD. Weighted multivariable logistic regression models and generalized additive models were used to investigate the independent relationships between NLR levels with DKD, albuminuria, and low-eGFR. Additionally, we examined the relationships between DKD, albuminuria, and low-eGFR with other inflammatory markers, such as the aggregate index of systemic inflammation (AISI), systemic immune-inflammation index (SII), system inflammation response index (SIRI), and platelet-to-lymphocyte ratio (PLR) and monocyte-to-lymphocyte ratio (MLR). Their diagnostic capabilities were evaluated and contrasted using receiver operating characteristic (ROC) curves.

**Results:**

44.65% of the 7,153 participants who were recruited for this study were males. DKD, albuminuria, and low-eGFR were prevalent in 31.76%, 23.08%, and 14.55% of cases, respectively. Positive correlations were seen between the NLR with the prevalences of DKD, albuminuria, and low-eGFR. Subgroup analysis and interaction tests revealed that the associations of NLR with DKD, albuminuria, and low-eGFR were not significantly different across populations. In addition, MLR, SII and SIRI showed positive associations with the prevalence of DKD. ROC analysis discovered that when compared to other inflammatory markers (MLR, PLR, SII, SIRI, and AISI), NLR may demonstrate more discriminatory power and accuracy in assessing the risk of DKD, albuminuria, and low-eGFR.

**Conclusion:**

Compared to other inflammatory markers (MLR, PLR, SII, SIRI, and AISI), NLR may serve as the more effective potential inflammatory marker for identifying the risk of DKD, albuminuria, and low-eGFR in US T2DM patients. T2DM patients with elevated levels of NLR, MLR, SII, and SIRI should be closely monitored for their potential risk to renal function.

## 1 Introduction

Diabetic kidney disease (DKD), which accounts for more than 50% of all instances of end-stage kidney disease (ESKD), has emerged as the most prevalent chronic kidney disease (CKD) worldwide due to the increased prevalence of type 2 diabetes mellitus (T2DM) associated with obesity (1–3). Even in the early stages of DKD, patients are more susceptible to cardiovascular illness (4). Recent studies have revealed that people with DKD have a greater risk of dying after developing coronavirus disease 2019 (COVID-19) (5). Thus, early intervention is necessary to stop DKD from progressing. Previous studies have identified inflammation, obesity, hypertension, smoking, and sex as significant risk factors for DKD (6–8). Among these, inflammation has drawn interest as a modifiable risk factor that may offer preventative options.

Chronic inflammation is considered a potential mechanism underlying DKD (9–11). However, the use of many inflammatory markers in routine clinical practice has been limited due to their cost and measurement-related technical problems. It is notable that the neutrophil-to-lymphocyte ratio (NLR), a laboratory index that is simple to measure and reasonably priced and is derived from routinely analyzed leukocyte characteristics, integrates the detrimental effects of neutrophils on endothelial damage with the antiatherosclerotic function of lymphocytes (12). In light of this, the NLR has been regarded as a practical biomarker of systemic inflammation (13, 14). The relationship between NLR and DKD has been researched in earlier studies. NLR levels were discovered by Wan et al. to be positively correlated with ACR levels and the prevalence of DKD in Chinese diabetic patients (15). NLR has been reported to be a reliable predictor of early DKD in a prospective study from Egypt (16). In a cohort of Japanese diabetics, higher NLR levels were connected to a higher frequency of albuminuria (17). To our knowledge, no study has examined how NLR is related to DKD in the US diabetic population.

Therefore, this study aims to examine this association between NLR and DKD using data from the National Health and Nutrition Examination Survey (NHANES) in adult T2DM patients in the United States.

## 2 Methods

### 2.1 Study population and participants selected

NHANES, conducted by the National Center for Health Statistics (NCHS), provides valuable cross-sectional data for research purposes (18). It is used to assess the physical and nutritional well-being of the non-institutionalized population in the US. Every two years, the NHANES survey data are updated to ensure that it is always up to date. Utilizing a stratified multi-stage probabilistic technique, the NHANES study design produces a sizable representative sample of enrolled individuals. The protocols for the NHANES survey have been approved by the NCHS research ethics review committee, and all study participants have provided informed consent. For more comprehensive information regarding the planning and execution of NHANES, please refer to the official NHANES website.

**Figure 1:**
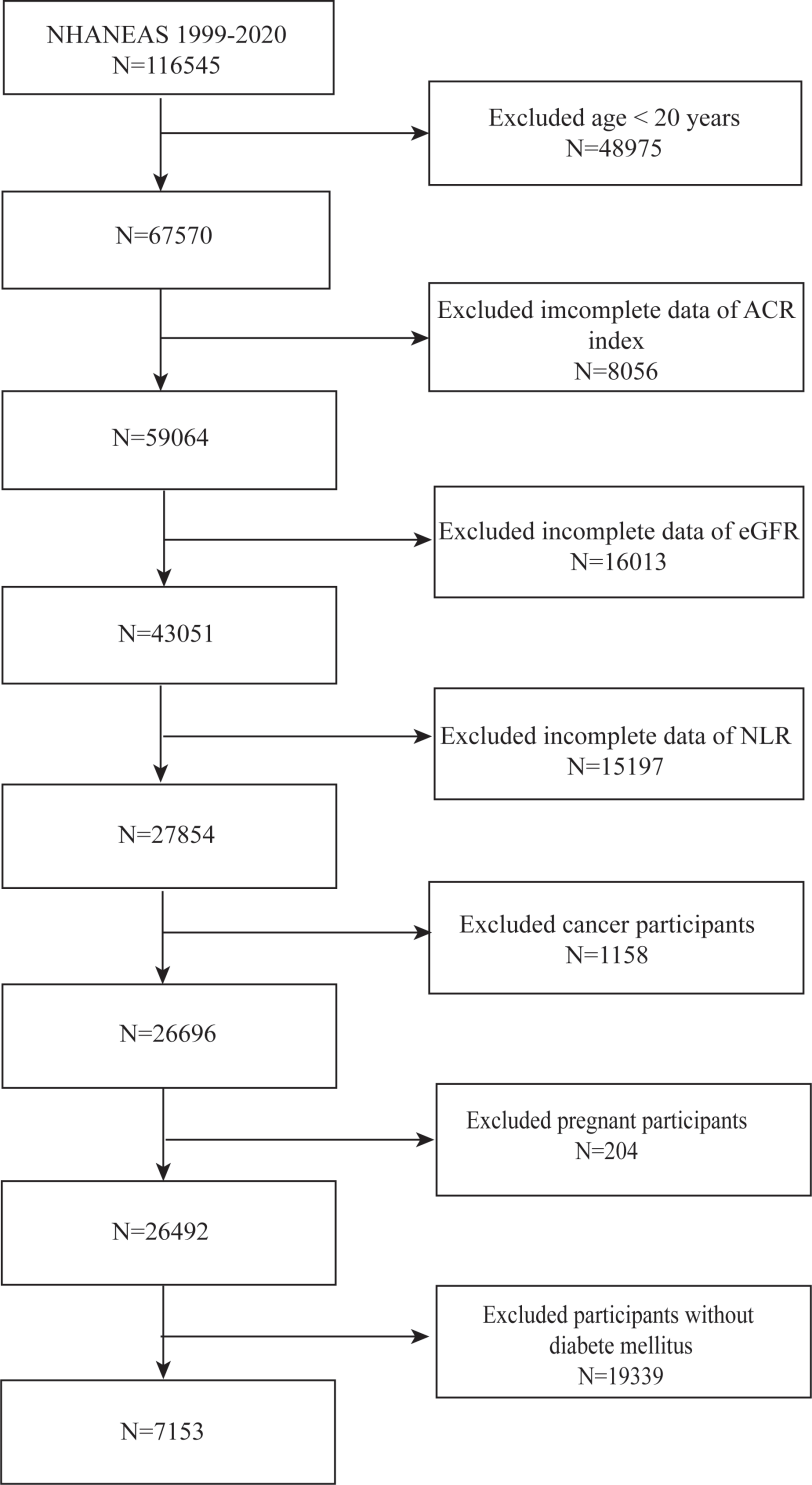
Flowchart of the sample selection from NHANES 1999–2020.

We utilized data from NHANES spanning the years 1999 to 2020 to select participants for our study. We eliminated those under the age of 20 (n = 48,975), those with cancer (n = 1,158), those in pregnancy (n = 204), and those lacking information on ACR (n = 8,506), eGFR (n = 16,013), and NLR (n = 15,197) after a stringent selection process. Furthermore, those without T2DM (n = 19,339) were not included in the study. After applying these exclusion criteria, our final cohort consisted of 7,153 eligible subjects (**Figure 1**).

### 2.2 Definition of NLR

Venous blood samples were collected in the morning after a fasting period to conduct routine clinical chemistry analysis. Using the Coulter counter method, we obtained the neutrophil count (NC) and lymphocyte count (LC) from the whole blood. The ratio of NC to LC was then used to calculate the NLR (19). We also looked at the associations between DKD and other inflammatory markers, such as the platelet-to-lymphocyte ratio (PLR) (PLR= platelet count(PC)/LC), monocyte-to-lymphocyte ratio (MLR) (MLR= monocyte count(MC)/LC), systemic immune-inflammation index (SII) (SII=PC * NC/LC), system inflammation response index (SIRI) (SIRI=NC * MC/LC), and aggregate index of systemic inflammation (AISI) (AISI=NC * PC * MC/LC), to fully assess the relationship between NLR and DKD.

### 2.3 Definition of DKD, low-eGFR, and albuminuria

Self-reported diabetes, the use of insulin or other diabetes drugs, or specific criteria based on fasting glucose (mmol/l) ≥ 7.0 or glycosylated hemoglobin A1c (HbA1c) (%) > 6.5 were all required for the diagnosis of diabetes. DKD was diagnosed with the low estimated glomerular filtration rate (eGFR) (< 60 mL/min/1.73 m^2^) or albuminuria (urinary albumin-to-creatinine ratio (ACR) ≥ 30 mg/g) in T2DM patients (20). The eGFR was calculated using the Chronic Kidney Disease Epidemiology Collaboration (CKD-EPI) equation for standardized creatinine (21). The main outcome variables in this study were albuminuria, low-eGFR, and DKD.

### 2.4 Covariates

Demographic parameters included sex, age, race, and education level. Additionally, we considered various anthropometric and laboratory covariates, such as body mass index (BMI)(normal weight, overweight, and obese), smoking status (≥100 cigarettes lifetime/<100 cigarettes lifetime), alcohol consumption (days) (number of days of alcohol consumption in the past year), total cholesterol (TC), high-density lipoprotein cholesterol (HDL-C), low-density lipoprotein cholesterol (LDL-C), triglycerides, aspartate aminotransferase (AST), alanine aminotransferase (ALT), family income to poverty ratio (PIR) (categorized as low income (≤1.3), medium income (>1.3 to 3.5), and high income (>3.5)), cardiovascular diseases (CVD), fasting glucose, glycohemoglobin, and marital status (married/never married/living with a partner/others, including widowed, divorced, or separated). DM-related treatment included insulin use and diabetes drug use.

In addition, we used hypertension in our study to account for variations in health status. A three-part criterion was used to define hypertension. Hypertension was defined as long as one of these criteria was met. Participants were asked to self-report their hypertension in the first segment using the question “Ever told you had hypertension”. The second segment is mean systolic blood pressure (SBP) ≥ 130 mmHg and/or mean diastolic blood pressure (DBP) ≥ 80 mmHg (22). The third segment employed the item “taking hypertension prescription” program to identify hypertensive participants Visit the webpage at www.cdc.gov/nchs/nhanes/ to learn more detailed information on these variables.

### 2.5 Statistical analysis

In all of our statistical analyses, we took into consideration the intricate sample design of a multi-stage cluster survey by the advice of the U.S. Centers for Disease Control and Prevention (CDC) (23). While categorical variables were shown as percentages, continuous variables were summarized using the mean and standard deviation. We used weighted t-tests or chi-square tests to evaluate differences between NLR (tertiles). We used weighted multivariable regression models in three distinct models to examine the relationships between NLR with DKD, albuminuria, and low-eGFR. Model 1 had no covariate adjustments. Model 2 included covariate adjustments for age, race, and sex. In Model 3, we adjusted for several covariates, including sex, age, race, education level, BMI, smoking status, alcohol consumption, TC, LDL-C, HDL-C, AST, ALT, triglycerides, PIR, CVD, hypertension, fasting glucose, glycohemoglobin, insulin use, diabetes drug use, and marital status. We conducted a sensitivity analysis by changing NLR from a continuous variable to a categorical variable (tertiles) to assess the robustness of our findings. Generalized additive models (GAM) and smooth curve fitting were used to address non-linear relationships. By fitting a two-segment linear regression model (segmented regression model) to each interval and comparing them to the one-line model (non-segmented) using the log-likelihood ratio test, we also looked at threshold effects. We utilized a two-step recursive method to find breakpoints. Using stratified multivariable logistic regression models stratified by sex, age, BMI, hypertension, and CVD, subgroup analysis was performed to examine the relationships between NLR with DKD, albuminuria, and low-eGFR. These stratification features were taken into consideration as potential effect modifiers. To examine the heterogeneity of relationships between the subgroups, an interaction term was also included. Furthermore, we used receiver operating characteristic (ROC) curves to evaluate the identifiable power of NLR and other inflammatory markers (MLR, PLR, SII, SIRI, and AISI) for DKD, albuminuria, and low-eGFR and compared the area under the curve (AUC) values. Missing values for categorical variables were handled using mode imputation, whereas missing values for continuous variables were handled by median imputation. We used the Empower software package and R version 4.1.3 to carry out all of our statistical studies. A two-tailed *p*-value < 0.05 was used to determine statistical significance.

## 3 Results

### 3.1 Participants characteristics at baseline

7,153 people in all, with a mean age of 48.91 ± 18.23 years and 44.65% males and 55.35% females, were included in our study. The prevalences of DKD, albuminuria, and low-eGFR were found to be 31.76%, 23.08%, and 14.55%, respectively. The mean NLR observed among the participants was 2.19 ± 1.34. The study found that individuals in the upper tertiles of NLR had higher prevalences of low-eGFR, albuminuria, and DKD than those in the lower tertiles (all *p* < 0.05) (**Table 1**).

**Table 1:** Baseline characteristics according to NLR tertiles.

NLR	Overall	Tertile 1	Tertile 2	Tertile 3	*P-*value
		(0.09–1.65)	(1.65–2.25)	(2.25–24.60)	
N	7153	2373	2386	2394	
NLR	2.19 ± 1.34	1.15 ± 0.28	1.93 ± 0.22	3.48 ± 1.58	<0.001
MLR	0.27 ± 0.12	0.19 ± 0.07	0.25 ± 0.07	0.36 ± 0.14	<0.001
PLR	121.75 ± 49.01	97.29 ± 36.29	118.30 ± 37.07	149.44 ± 55.96	<0.001
SII	551.22 ± 394.88	289.27 ± 113.20	481.10 ± 155.28	823.44 ± 516.05	<0.001
SIRI	1.24 ± 0.98	0.69 ± 0.29	1.06 ± 0.40	2.05 ± 1.23	<0.001
AISI	321.81 ± 287.59	162.26 ± 92.55	280.32 ± 145.53	522.34 ± 388.62	<0.001
Age, years					0.072
20-40	2736 (38.25%)	946 (39.87%)	915 (38.35%)	875 (36.55%)	
41-60	2243 (31.36%)	751 (31.65%)	740 (31.01%)	752 (31.41%)	
>60	2174 (30.39%)	676 (28.49%)	731 (30.64%)	767 (32.04%)	
Sex, n (%)					0.001
Male	3194 (44.65%)	1130 (47.62%)	1043 (43.71%)	1021 (42.65%)	
Female	3959 (55.35%)	1243 (52.38%)	1343 (56.29%)	1373 (57.35%)	
Race, n (%)					0.320
Mexican American	1242 (17.36%)	432 (18.20%)	419 (17.56%)	391 (16.33%)	
Other Hispanic	663 (9.27%)	214 (9.02%)	239 (10.02%)	210 (8.77%)	
Non-Hispanic White	3171 (44.33%)	1066 (44.92%)	1020 (42.75%)	1085 (45.32%)	
Non-Hispanic Black	1418 (19.82%)	443 (18.67%)	485 (20.33%)	490 (20.47%)	
Other Races	659 (9.21%)	218 (9.19%)	223 (9.35%)	218 (9.11%)	
Education level, n (%)					0.902
Less than high school	1893 (26.46%)	623 (26.25%)	650 (27.24%)	620 (25.90%)	
High school or GED	1586 (22.17%)	534 (22.50%)	519 (21.75%)	533 (22.26%)	
Above high school	3653 (51.07%)	1211 (51.03%)	1209 (50.67%)	1233 (51.50%)	
Others	21 (0.29%)	5 (0.21%)	8 (0.34%)	8 (0.33%)	
Marital status, n (%)					0.112
Married	3210 (52.73%)	1103 (54.31%)	1052 (51.47%)	1055 (52.41%)	
Never married	1124 (18.46%)	372 (18.32%)	384 (18.79%)	368 (18.28%)	
Living with a partner	477 (7.84%)	171 (8.42%)	147 (7.19%)	159 (7.90%)	
Others	1277 (20.98%)	385 (18.96%)	461 (22.55%)	431 (21.41%)	
BMI, n (%)					0.010
Normal weight	1891 (26.74%)	653 (27.75%)	661 (28.03%)	577 (24.43%)	
Overweight	2188 (30.93%)	746 (31.70%)	716 (30.36%)	726 (30.74%)	
Obese	2994 (42.33%)	954 (40.54%)	981 (41.60%)	1059 (44.83%)	
Smoking status, n (%)					< 0.001
≥100 cigarettes lifetime	3067 (47.05%)	1007 (46.79%)	1001 (46.41%)	1059 (47.92%)	
< 100 cigarettes lifetime	3452 (52.95%)	457 (19.26%)	781 (32.73%)	1034 (43.19%)	
PIR, n (%)					<0.001
Low income	2708 (40.99%)	449 (20.43%)	531 (24.08%)	582 (26.42%)	
Medium income	2336 (35.36%)	728 (33.12%)	756 (34.29%)	852 (38.67%)	
High income	1562 (23.65%)	1021 (46.45%)	918 (41.63%)	769 (34.91%)	
Insulin use, n (%)					<0.001
Yes	1360 (21.97%)	372 (18.34%)	428 (20.79%)	560 (26.64%)	
No	4829 (78.03%)	1656 (81.66%)	1631 (79.21%)	1542 (73.36%)	
Diabetes drug use, n (%)					0.007
Yes	3810 (69.56%)	1252 (70.10%)	1308 (71.67%)	1250 (66.99%)	
No	1667 (30.44%)	534 (29.90%)	517 (28.33%)	616 (33.01%)	
CVD, n (%)	643 (8.99%)	141 (5.94%)	210 (8.80%)	292 (12.20%)	< 0.001
Alcohol consumption, days	4.09 ± 8.85	3.81 ± 3.75	4.42 ± 13.62	4.04 ± 5.61	0.346
Hypertension, n (%)	4423 (61.83%)	1396 (58.83%)	1460 (61.19%)	1567 (65.46%)	< 0.001
Fasting glucose, mmol/L	8.91 ± 3.62	8.73 ± 3.62	9.14 ± 3.81	8.86 ± 3.43	0.022
glycohemoglobin, %	7.44 ± 1.81	7.46 ± 1.83	7.53 ± 1.83	7.34 ± 1.75	0.001
TC, mg/dL	182.81 ± 43.78	183.39 ± 43.78	182.46 ± 42.51	182.59 ± 45.02	0.732
HDL-C, mg/dL	52.11 ± 15.93	53.20 ± 15.72	51.30 ± 15.37	51.83 ± 16.62	<0.001
LDL-C, mg/dL	105.25 ± 35.46	106.38 ± 36.71	104.44 ± 33.88	104.89 ± 35.71	0.228
ALT, U/L	24.39 ± 19.38	24.83 ± 20.81	24.28 ± 18.74	24.06 ± 18.51	0.408
AST, U/L	24.94 ± 17.39	25.34 ± 23.31	24.77 ± 12.83	24.72 ± 14.12	0.437
Triglyceride, mg/dL	121.94 ± 114.70	115.33 ± 118.14	126.01 ± 122.55	124.66 ± 101.56	0.009
ACR, mg/g	90.98 ± 488.64	54.06 ± 337.31	97.52 ± 473.45	121.07 ± 612.47	<0.001
Albuminuria, n (%)	1651 (23.08%)	321 (13.53%)	585 (24.52%)	745 (31.12%)	< 0.001
eGFR, mL/min/1.73 m2	90.55 ± 29.84	94.51 ± 24.80	90.18 ± 29.79	87.00 ± 33.75	<0.001
Low-eGFR, n (%)	1041 (14.55%)	216 (9.10%)	346 (14.50%)	479 (20.01%)	< 0.001
DKD, n (%)	2272 (31.76%)	457 (19.26%)	781 (32.73%)	1034 (43.19%)	< 0.001

NLR, neutrophil-to-lymphocyte ratio; MLR, monocyte-to-lymphocyte ratio; PLR, platelet-to-lymphocyte ratio; SII, systemic immune-inflammation index; SIRI, system inflammation response index; AISI, aggregate index of systemic inflammation; GED, general educational development; BMI, body mass index; PIR, family income to poverty ratio; CVD, cardiovascular diseases; TC, total cholesterol; HDL-C, high-density lipoprotein-cholesterol; LDL-C, low-density lipoprotein cholesterol; ALT: alanine aminotransferase; AST: aspartate aminotransferase; ACR, urinary albumin-to-creatinine ratio; eGFR, urinary albumin-to-creatinine ratio; DKD, diabetic kidney disease.

Additionally, we found significant variations in several variables across the NLR tertiles, including sex, BMI, HDL-C, triglycerides, PIR, fasting glucose, glycohemoglobin, insulin use, diabetes drug use, smoking status, CVD, hypertension, ACR, eGFR, MLR, PLR, SII, SIRI, and AISI (all *p* < 0.05) (**Table 1**).

### 3.2 Association between NLR and DKD

The correlations between NLR and other inflammatory markers with DKD were examined and presented in **Table 2**. In Model 3, NLR, MLR, SII, and SIRI were significantly positively correlated with DKD (NLR: OR = 2.90; 95% CI: 1.51, 5.58; MLR: OR = 6.93; 95% CI: 2.37, 20.31; SII: OR = 1.00; 95% CI: 1.00, 1.01; SIRI: OR = 3.01; 95% CI: 1.18, 7.68). Significant associations remained even when the inflammatory markers were categorized into tertiles. Individuals in the highest tertiles of NLR, MLR, and SIRI exhibited higher prevalences of DKD compared to those in the lowest tertile (all *p* for trend < 0.05).

**Table 2:** Associations between NLR and other inflammatory markers with DKD, albuminuria, and low-eGFR.

Index	Outcome	Continuous or categories	Model 1^3^		Model 2^4^		Model 3^5^	
			OR^1^ (95%CI^2^)	*P-* value	OR (95%CI)	*P-* value	OR (95%CI)	*P-* value
**NLR**	**DKD**	NLR as continuous variable	1.45 (1.38, 1.52)	<0.0001	1.44 (1.37, 1.51)	<0.0001	2.90 (1.51, 5.58)	0.0014
		Tertile 1	Reference		Reference		Reference	
		Tertile 2	2.04 (1.79, 2.33)	<0.0001	2.01 (1.76, 2.30)	<0.0001	2.16 (0.56, 8.30)	0.2632
		Tertile 3	3.19 (2.80, 3.63)	<0.0001	3.12 (2.74, 3.56)	<0.0001	6.59 (1.72, 25.25)	0.0059
		*P* for trend	<0.0001		<0.0001		0.0057	
	**Albuminuria**	NLR as continuous variable	1.36 (1.29, 1.43)	<0.0001	1.36 (1.30, 1.43)	<0.0001	2.01 (1.10, 3.68)	0.0224
		Tertile 1	Reference		Reference		Reference	
		Tertile 2	2.08 (1.79, 2.41)	<0.0001	2.09 (1.80, 2.43)	<0.0001	7.13 (1.18, 43.14)	0.0324
		Tertile 3	2.89 (2.50, 3.34)	<0.0001	2.92 (2.52, 3.38)	<0.0001	7.31 (1.43, 37.34)	0.0169
		*P* for trend	<0.0001		<0.0001		0.0275	
	**Low-eGFR**	NLR as continuous variable	1.25 (1.18, 1.31)	<0.0001	1.23 (1.17, 1.30)	<0.0001	2.50 (1.05, 5.99)	0.0390
		Tertile 1	Reference		Reference		Reference	
		Tertile 2	1.69 (1.41, 2.03)	<0.0001	1.63 (1.35, 1.96)	<0.0001	0.50 (0.08, 3.20)	0.4622
		Tertile 3	2.50 (2.10, 2.97)	<0.0001	2.36 (1.98, 2.82)	<0.0001	2.31 (0.49, 10.88)	0.2899
		*P* for trend	<0.0001		<0.0001		0.2920	
**MLR**	**DKD**	MLR as continuous variable	9.45 (6.14, 14.54)	<0.0001	8.84 (5.73, 13.65)	<0.0001	6.93 (2.37, 20.31)	0.0004
		Tertile 1	Reference		Reference		Reference	
		Tertile 2	1.57 (1.38, 1.79)	<0.0001	1.56 (1.37, 1.78)	<0.0001	3.17 (0.91, 11.01)	0.0688
		Tertile 3	2.17 (1.92, 2.46)	<0.0001	2.13 (1.88, 2.41)	<0.0001	5.73 (1.46, 22.50)	0.0124
		*P* for trend	<0.0001		<0.0001		0.0127	
	**Albuminuria**	MLR as continuous variable	6.96 (4.44, 10.90)	<0.0001	7.14 (4.55, 11.19)	<0.0001	5.75 (1.76, 18.76)	0.0038
		Tertile 1	Reference		Reference		Reference	
		Tertile 2	1.47 (1.27, 1.69)	<0.0001	1.47 (1.28, 1.70)	<0.0001	2.11 (0.45, 9.95)	0.3435
		Tertile 3	1.90 (1.65, 2.18)	<0.0001	1.91 (1.67, 2.20)	<0.0001	7.96 (1.33, 47.76)	0.0232
		*P* for trend	<0.0001		<0.0001		0.0212	
	**Low-eGFR**	MLR as continuous variable	4.47 (2.69, 7.41)	<0.0001	3.86 (2.28, 6.55)	<0.0001	2.99 (0.76, 11.83)	0.1177
		Tertile 1	Reference		Reference		Reference	
		Tertile 2	1.41 (1.19, 1.68)	<0.0001	1.38 (1.16, 1.65)	0.0004	2.54 (0.42, 15.21)	0.3074
		Tertile 3	1.86 (1.58, 2.20)	<0.0001	1.76 (1.49, 2.09)	<0.0001	2.84 (0.45, 17.99)	0.2665
		*P* for trend	<0.0001		<0.0001		0.3008	
**PLR**	**DKD**	PLR as continuous variable	1.00 (1.00, 1.01)	<0.0001	1.00 (1.00, 1.01)	<0.0001	1.01 (0.99, 1.02)	0.2460
		Tertile 1	Reference		Reference		Reference	
		Tertile 2	1.26 (1.11, 1.42)	0.0004	1.26 (1.11, 1.43)	0.0004	4.46 (1.19, 16.71)	0.0267
		Tertile 3	1.58 (1.40, 1.79)	<0.0001	1.58 (1.40, 1.79)	<0.0001	2.40 (0.67, 8.67)	0.1807
		*P* for trend	<0.0001		<0.0001		0.2829	
	**Albuminuria**	PLR as continuous variable	1.00 (1.00, 1.01)	<0.0001	1.00 (1.00, 1.01)	<0.0001	1.01 (0.99, 1.03)	0.1251
		Tertile 1	Reference		Reference		Reference	
		Tertile 2	1.21 (1.05, 1.39)	0.0078	1.21 (1.05, 1.39)	0.0073	2.36 (0.42, 13.18)	0.3271
		Tertile 3	1.46 (1.27, 1.67)	<0.0001	1.46 (1.27, 1.67)	<0.0001	2.54 (0.50, 12.93)	0.2599
		*P* for trend	<0.0001		<0.0001		0.3157	
	**Low-eGFR**	PLR as continuous variable	1.00 (1.00, 1.01)	0.0006	1.00 (1.00, 1.01)	0.0002	1.01 (0.99, 1.02)	0.4864
		Tertile 1	Reference		Reference		Reference	
		Tertile 2	1.24 (1.05, 1.46)	0.0126	1.25 (1.05, 1.49)	0.0121	5.64 (0.77, 41.39)	0.0890
		Tertile 3	1.49 (1.27, 1.76)	<0.0001	1.51 (1.27, 1.78)	<0.0001	2.07 (0.27, 15.68)	0.4817
		*P* for trend	<0.0001		<0.0001		0.6258	
**SII**	**DKD**	SII as continuous variable	1.00 (1.00, 1.01)	<0.0001	1.00 (1.00, 1.01)	<0.0001	1.00 (1.00, 1.01)	0.0426
		Tertile 1	Reference		Reference		Reference	
		Tertile 2	1.69 (1.49, 1.92)	<0.0001	1.68 (1.48, 1.91)	<0.0001	1.17 (0.37, 3.75)	0.7860
		Tertile 3	2.00 (1.76, 2.27)	<0.0001	1.98 (1.74, 2.25)	<0.0001	1.65 (0.47, 5.80)	0.4330
		*P* for trend	<0.0001		<0.0001		0.4279	
	**Albuminuria**	SII as continuous variable	1.00 (1.00, 1.01)	<0.0001	1.00 (1.00, 1.01)	<0.0001	1.00 (0.99, 1.00)	0.1171
		Tertile 1	Reference		Reference		Reference	
		Tertile 2	1.65 (1.44, 1.90)	<0.0001	1.66 (1.44, 1.91)	<0.0001	1.20 (0.27, 5.45)	0.8112
		Tertile 3	1.83 (1.60, 2.11)	<0.0001	1.85 (1.60, 2.12)	<0.0001	1.51 (0.32, 7.11)	0.6030
		*P* for trend	<0.0001		<0.0001		0.6030	
	**Low-eGFR**	SII as continuous variable	1.00 (1.00, 1.01)	<0.0001	1.00 (1.00, 1.01)	<0.0001	1.00 (0.99, 1.01)	0.0925
		Tertile 1	Reference		Reference		Reference	
		Tertile 2	1.56 (1.32, 1.86)	<0.0001	1.54 (1.29, 1.84)	<0.0001	2.05 (0.35, 11.94)	0.4261
		Tertile 3	1.79 (1.52, 2.12)	<0.0001	1.76 (1.48, 2.09)	<0.0001	1.11 (0.18, 6.79)	0.9081
		*P* for trend	<0.0001		<0.0001		0.9967	
**SIRI**	**DKD**	SIRI as continuous variable	1.31 (1.23, 1.39)	<0.0001	1.30 (1.22, 1.38)	<0.0001	3.01 (1.18, 7.68)	0.0213
		Tertile 1	Reference		Reference		Reference	
		Tertile 2	1.76 (1.55, 2.01)	<0.0001	1.74 (1.53, 1.98)	<0.0001	2.40 (0.63, 9.16)	0.1984
		Tertile 3	2.26 (1.99, 2.57)	<0.0001	2.20 (1.94, 2.50)	<0.0001	4.41 (1.13, 17.18)	0.0325
		*P* for trend	<0.0001		<0.0001		0.0378	
	**Albuminuria**	SIRI as continuous variable	1.25 (1.17, 1.32)	<0.0001	1.25 (1.18, 1.33)	<0.0001	2.45 (0.92, 6.57)	0.0742
		Tertile 1	Reference		Reference		Reference	
		Tertile 2	1.76 (1.53, 2.03)	<0.0001	1.77 (1.53, 2.04)	<0.0001	1.82 (0.36, 9.06)	0.4663
		Tertile 3	2.11 (1.83, 2.43)	<0.0001	2.13 (1.85, 2.46)	<0.0001	3.84 (0.87, 17.02)	0.0767
		*P* for trend	<0.0001		<0.0001		0.0764	
	**Low-eGFR**	SIRI as continuous variable	1.17 (1.10, 1.25)	<0.0001	1.14 (1.07, 1.22)	<0.0001	2.17 (0.61, 7.77)	0.2320
		Tertile 1	Reference		Reference		Reference	
		Tertile 2	1.45 (1.22, 1.72)	<0.0001	1.40 (1.17, 1.68)	0.0002	1.29 (0.19, 8.93)	0.7985
		Tertile 3	1.82 (1.54, 2.15)	<0.0001	1.68 (1.42, 2.00)	<0.0001	1.20 (0.21, 7.01)	0.8357
		*P* for trend	<0.0001		<0.0001		0.8698	
**AISI**	**DKD**	AISI as continuous variable	1.00 (1.00, 1.01)	<0.0001	1.00 (1.00, 1.01)	<0.0001	1.00 (0.99, 1.01)	0.1370
		Tertile 1	Reference		Reference		Reference	
		Tertile 2	1.49 (1.32, 1.69)	<0.0001	1.47 (1.29, 1.66)	<0.0001	0.86 (0.25, 3.01)	0.8196
		Tertile 3	1.55 (1.37, 1.75)	<0.0001	1.51 (1.34, 1.72)	<0.0001	1.96 (0.54, 7.08)	0.3032
		*P* for trend	<0.0001		<0.0001		0.2432	
	**Albuminuria**	AISI as continuous variable	1.00 (1.00, 1.01)	<0.0001	1.00 (1.00, 1.01)	<0.0001	1.00 (0.99, 1.01)	0.1744
		Tertile 1	Reference		Reference		Reference	
		Tertile 2	1.43 (1.25, 1.64)	<0.0001	1.44 (1.25, 1.65)	<0.0001	1.31 (0.29, 5.92)	0.7254
		Tertile 3	1.50 (1.30, 1.72)	<0.0001	1.50 (1.31, 1.73)	<0.0001	2.00 (0.46, 8.72)	0.3547
		*P* for trend	<0.0001		<0.0001		0.3493	
	**Low-eGFR**	AISI as continuous variable	1.00 (1.00, 1.01)	0.0081	1.00 (1.00, 1.01)	0.0353	1.00 (0.99, 1.01)	0.3983
		Tertile 1	Reference		Reference		Reference	
		Tertile 2	1.37 (1.16, 1.62)	0.0002	1.32 (1.12, 1.57)	0.0013	0.47 (0.06, 3.59)	0.4705
		Tertile 3	1.40 (1.19, 1.65)	<0.0001	1.32 (1.11, 1.56)	0.0016	0.57 (0.08, 4.10)	0.5780
		*P* for trend	<0.0001		0.0070		0.6994	

In sensitivity analysis, NLR, MLR, PLR, SII, SIRI, and AISI were converted from continuous variables to categorical variables (tertiles).

^1^OR: Odd ratio.

^2^95% CI: 95% confidence interval.

^3^Model 1: No covariates were adjusted.

^4^Model 2: Adjusted for age, sex, and race.

^5^Model 3: Adjusted for sex, age, race, education level, BMI, smoking status, alcohol consumption, TC, LDL-C, HDL-C, AST, ALT, triglycerides, PIR, CVD, hypertension, fasting glucose, glycohemoglobin, insulin use, diabetes drug use, and marital status.

**Figure 2:**
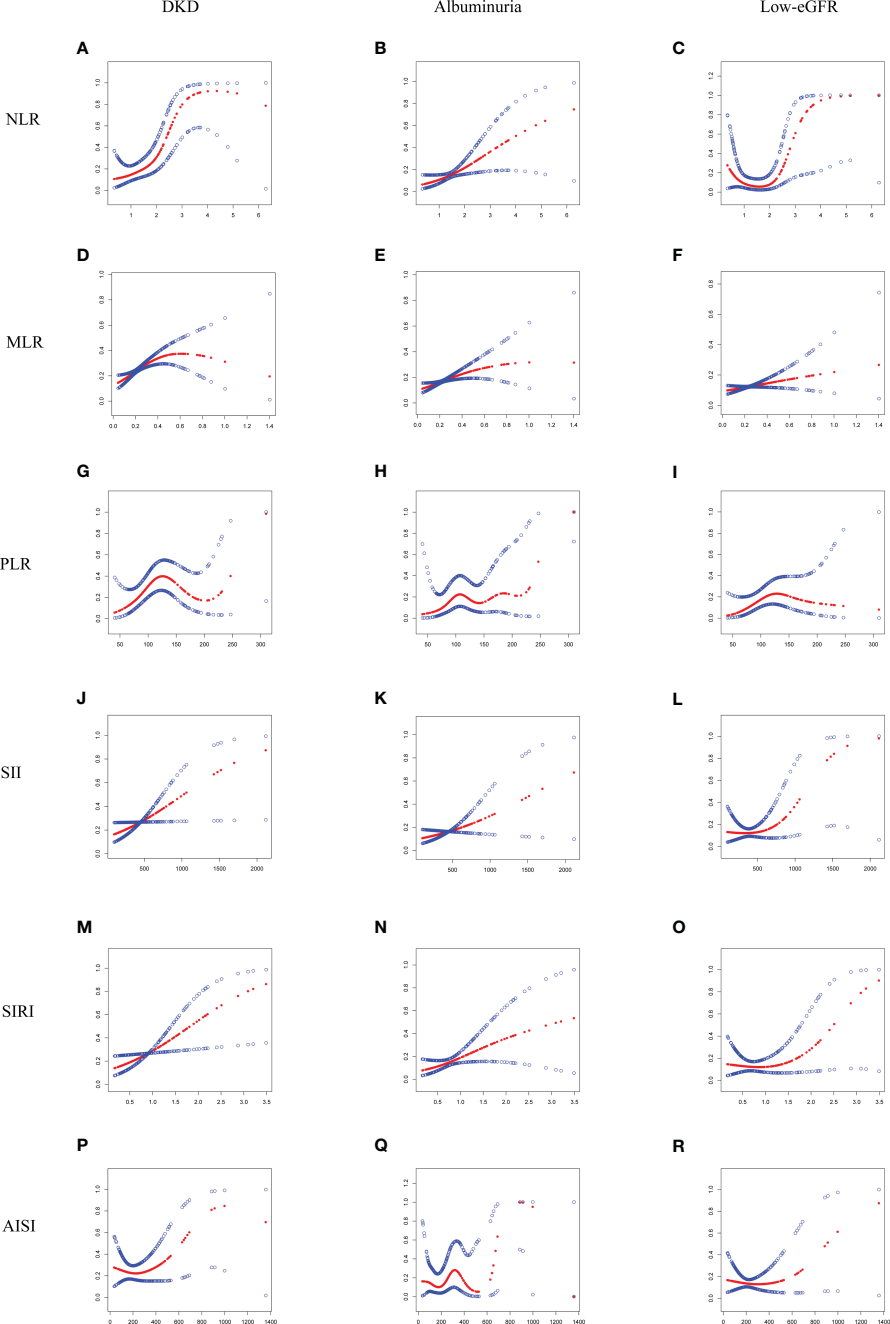
Smooth curve fitting for NLR and other inflammatory markers with DKD, albuminuria, and low-eGFR. **(A)** NLR and DKD; **(B)** NLR and albuminuria; **(C)** NLR and low-eGFR; **(D)** MLR and DKD; **(E)** MLR and albuminuria; **(F)** MLR and low-eGFR; **(G)** PLR and DKD; **(H)** PLR and albuminuria; **(I)** PLR and low-eGFR; **(J)** SII and DKD; **(K)** SII and albuminuria; **(L)** SII and low-eGFR; **(M)** SIRI and DKD; **(N)** SIRI and albuminuria; **(O)** SIRI and low-eGFR; **(P)** AISI and DKD; **(Q)** AISI and albuminuria; **(R)** AISI and low-eGFR.

The threshold effect of the nonlinear relationship between MLR and DKD was found using GAM and smooth curve fitting, and it was shown to have a breakpoint of 0.43 (logarithmic likelihood ratio test P-value <0.05) (**Table 3**). The relationship between NLR and DKD was not shown to be nonlinear (**Figure 2**).

**Table 3:** Threshold effect analysis of NLR and other inflammatory markers on DKD, albuminuria, and low-eGFR using a two-piecewise linear regression model in Model 3.

	DKD		Albuminuria		Low-eGFR	
	OR^1^ (95%CI^2^)	*P-* value	OR (95%CI)	*P-* value	OR (95%CI)	*P-* value
NLR						
Fitting by standard linear model	2.90 (1.51, 5.58)	0.0014	2.01 (1.10, 3.68)	0.0224	2.50 (1.05, 5.99)	0.0390
Fitting by two-piecewise linear model						
Breakpoint (K)	3.43		3.22		1.88	
OR1(< K)	4.30 (1.77, 10.48)	0.0013	3.28 (1.30, 8.31)	0.0121	0.27 (0.04, 1.73)	0.1685
OR2(> K)	0.88 (0.14, 5.74)	0.8961	0.60 (0.10, 3.71)	0.5863	24.02 (2.72, 212.30)	0.0042
OR2/OR1	0.21 (0.02, 2.08)	0.1799	0.18 (0.02, 1.98)	0.1631	88.13 (2.72, 285.20)	0.0116
Logarithmic likelihood ratio test P-value	0.162		0.136		0.003	
MLR						
Fitting by standard linear model	6.93 (2.37, 20.31)	0.0004	5.75 (1.76, 18.76)	0.0038	2.99 (0.76, 11.83)	0.1177
Fitting by two-piecewise linear model						
Breakpoint (K)	0.43		0.29		0.21	
OR1(< K)	28.87 (6.52, 127.78)	<0.0001	55.20 (3.81, 799.07)	0.0033	72.06 (0.60, 455.06)	0.0747
OR2(> K)	0.22 (0.01, 4.60)	0.3281	1.47 (0.21, 10.20)	0.6974	1.34 (0.22, 8.33)	0.7512
OR2/OR1	0.01 (0.01, 0.34)	0.0115	0.03 (0.01, 1.25)	0.0647	0.01 (0.01, 5.49)	0.1468
Logarithmic likelihood ratio test P-value	0.006		0.060		0.138	
PLR						
Fitting by standard linear model	1.01 (0.99, 1.02)	0.2460	1.01 (0.99, 1.03)	0.1251	1.01 (0.99, 1.02)	0.4864
Fitting by two-piecewise linear model						
Breakpoint (K)	113.64		193.57		66.14	
OR1(< K)	1.03 (0.99, 1.06)	0.0770	1.00 (0.99, 1.02)	0.7448	14.14 (0.01, 52.10)	0.7752
OR2(> K)	1.00 (0.98, 1.01)	0.6753	1.06 (1.00, 1.12)	0.0337	0.99 (0.98, 1.01)	0.5739
OR2/OR1	0.97 (0.93, 1.01)	0.1409	1.06 (0.99, 1.13)	0.0714	0.01 (0.01, 2.74)	0.7750
Logarithmic likelihood ratio test P-value	0.129		0.058		0.003	
SII						
Fitting by standard linear model	1.00 (1.00, 1.01)	0.0426	1.00 (0.99, 1.00)	0.1171	1.00 (0.99, 1.01)	0.0925
Fitting by two-piecewise linear model						
Breakpoint (K)	149.52		850		245	
OR1(< K)	0.97 (0.92, 1.02)	0.2072	1.00 (0.99, 1.01)	0.1983	0.98 (0.96, 1.00)	0.0597
OR2(> K)	1.00 (1.00, 1.01)	0.0218	1.00 (0.99, 1.01)	0.7228	1.00 (1.00, 1.01)	0.0213
OR2/OR1	1.04 (0.98, 1.09)	0.1844	1.00 (0.98, 1.00)	0.6001	1.03 (1.00, 1.05)	0.0389
Logarithmic likelihood ratio test P-value	0.182		0.599		0.031	
SIRI						
Fitting by standard linear model	3.01 (1.18, 7.68)	0.0213	2.45 (0.92, 6.57)	0.0742	2.17 (0.61, 7.77)	0.2320
Fitting by two-piecewise linear model						
Breakpoint (K)	1.96		1.03		0.87	
OR1(< K)	4.88 (1.34, 17.81)	0.0165	17.66 (1.09, 286.46)	0.0434	0.02 (0.01, 2.56)	0.1176
OR2(> K)	0.68 (0.04, 12.59)	0.7940	0.97 (0.20, 4.68)	0.9721	12.02 (1.27, 113.42)	0.0299
OR2/OR1	0.14 (0.01, 5.03)	0.2811	0.06 (0.01, 2.38)	0.1312	94.21 (0.91, 374.26)	0.0537
Logarithmic likelihood ratio test P-value	0.265		0.121		0.039	
AISI						
Fitting by standard linear model	1.00 (0.99, 1.01)	0.1370	1.00 (0.99, 1.01)	0.1744	1.00 (0.99, 1.01)	0.3983
Fitting by two-piecewise linear model						
Breakpoint (K)	252.08		143.92		264.44	
OR1(< K)	1.00 (0.99, 1.00)	0.3813	0.99 (0.97, 1.02)	0.5628	0.99 (0.98, 1.00)	0.1627
OR2(> K)	1.00 (1.00, 1.01)	0.0427	1.00 (1.00, 1.01)	0.1238	1.01 (1.00, 1.01)	0.0572
OR2/OR1	1.01 (0.99, 1.02)	0.1642	1.01 (0.98, 1.04)	0.4599	1.01 (1.00, 1.03)	0.0810
Logarithmic likelihood ratio test P-value	0.156		0.460		0.070	

Adjusted for sex, age, race, education level, BMI, smoking status, alcohol consumption, TC, LDL-C, HDL-C, AST, ALT, triglycerides, PIR, CVD, hypertension, fasting glucose, glycohemoglobin, insulin use, diabetes drug use, and marital status.

^1^OR: Odd ratio.

^2^95% CI: 95% confidence interval.

### 3.3 Association between NLR and albuminuria

The study revealed that elevated levels of NLR and MLR were linked to an increased prevalence of albuminuria (**Table 2**). Model 3 demonstrated that each unit increase in NLR and MLR corresponded to a 1.01-fold and 4.75-fold rise in albuminuria prevalence, respectively (NLR: OR = 2.01; 95% CI: 1.10, 3.68; MLR: OR = 5.75; 95% CI: 1.76, 18.76). To further investigate the relationships, sensitivity analysis was conducted by categorizing inflammatory markers into tertiles. Participants who were in the higher tertiles of NLR showed a higher prevalence of albuminuria than those in the lower tertiles (*p* for trend < 0.05).

Additionally, through the use of GAM and smooth curve fitting, no significant nonlinear connections were discovered between NLR and other inflammatory markers with albuminuria (**Figure 2**; **Table 3**).

### 3.4 Association between NLR and low-eGFR

Three distinct models were used in the study to examine the connections between NLR and other inflammatory markers with low-eGFR (**Table 2**). In Model 3, a positive correlation remained only between NLR and low-eGFR, indicating that as each unit increased in NLR, the prevalence of low-eGFR increased by 1.50-fold (OR = 2.50; 95% CI:1.05, 5.99).

A nonlinear association with a calculated breakpoint of 1.88 was identified between NLR and low-eGFR through GAM and smooth curve fitting (logarithmic likelihood ratio test *P*-value < 0.05) (**Figure 2**). When NLR > 1.88, NLR displayed a positive connection with low-eGFR. There was no significant connection seen on the left side of the breakpoint, nevertheless (**Table 3**).

### 3.5 Subgroup analysis

**Figure 3:**
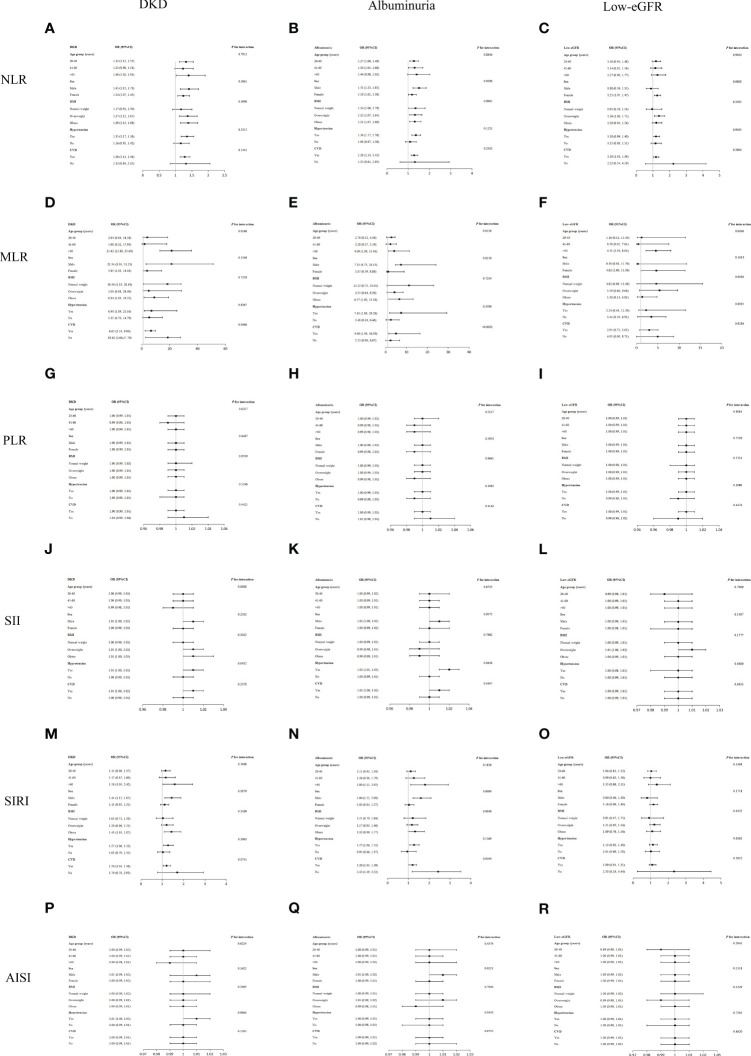
Subgroup analysis for the associations of NLR and other inflammatory markers with DKD, albuminuria, and low-eGFR. **(A)** NLR and DKD; **(B)** NLR and albuminuria; **(C)** NLR and low-eGFR; **(D)** MLR and DKD; **(E)** MLR and albuminuria; **(F)** MLR and low-eGFR; **(G)** PLR and DKD; **(H)** PLR and albuminuria; **(I)** PLR and low-eGFR; **(J)** SII and DKD; **(K)** SII and albuminuria; **(L)** SII and low-eGFR; **(M)** SIRI and DKD; **(N)** SIRI and albuminuria; **(O)** SIRI and low-eGFR; **(P)** AISI and DKD; **(Q)** AISI and albuminuria; **(R)** AISI and low-eGFR.

Our findings imply that there was an inconsistent association between NLR and other inflammatory markers with DKD (**Figure 3**). Significant associations were found between NLR and DKD in all subgroups stratified by sex (all *p* < 0.05). In the 41-60 years old, normal weight, nonhypertensive, and nonCVD population, positive but nonsignificant associations were observed. The results of the interaction tests indicated that in each subgroup, the relationship between NLR and DKD was not significantly impacted by age, sex, BMI, hypertension, or CVD (all *p* for interaction > 0.05). Additionally, the association between MLR and DKD was dependent on age, particularly applicable to individuals over 60 years of age.

Regarding the associations between NLR and PLR with albuminuria, there was no substantial association observed across different population subgroups, indicating a consistent association across populations (all *p* for interaction > 0.05) (**Figure 3**).

Age, sex, BMI, hypertension, and CVD were found to have no significant impact on the correlations between low-eGFR with NLR, MLR, PLR, SII, SIRI, and AISI, according to the interaction tests (all *p* for interaction > 0.05) (**Figure 3**).

### 3.6 ROC analysis

**Figure 4:**
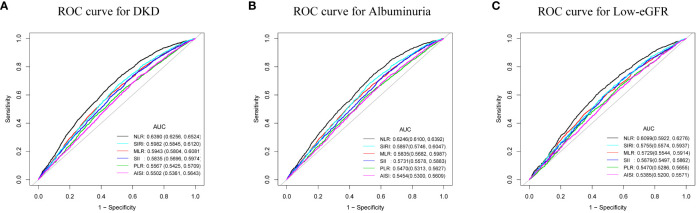
ROC curves and the AUC values of the six inflammatory markers (NLR, MLR, PLR, SII, SIRI, and AISI) in diagnosing DKD, albuminuria and low-eGFR. **(A)** Six inflammatory markers were assessed to identify DKD. **(B)** Six inflammatory markers were assessed to identify albuminuria. **(C)** Six inflammatory markers were assessed to identify low-eGFR.

The AUC values were calculated to assess the predictive accuracy of NLR and other inflammatory markers (MLR, PLR, SII, SIRI, and AISI) in predicting DKD, albuminuria, and low-eGFR (**Figure 4**). We observed that NLR had the highest AUC value among all inflammatory markers in predicting DKD, albuminuria, and low-eGFR. **Table 4** shows that there was a statistically significant difference in AUC values between NLR and other inflammatory markers (all *p* < 0.05). This suggests that when it comes to assessing the risk of DKD, albuminuria, and low-eGFR, NLR may be more accurate and discriminative than other inflammatory markers (MLR, PLR, SII, SIRI, and AISI).

**Table 4:** Comparison of AUC values between NLR and other inflammatory markers.

Test	AUC^1^	95%CI^2^ low	95%CI upp	Best threshold	Specificity	Sensitivity	*P* for different in AUC
DKD							
NLR	0.6390	0.6256	0.6524	1.3521	0.4913	0.7201	Reference
MLR	0.5943	0.5804	0.6081	0.2198	0.5269	0.6202	<0.0001
PLR	0.5567	0.5425	0.5709	109.1833	0.4811	0.6162	<0.0001
SII	0.5835	0.5696	0.5974	327.8316	0.3986	0.7377	<0.0001
SIRI	0.5982	0.5845	0.6120	0.7214	0.4911	0.6725	<0.0001
AISI	0.5502	0.5361	0.5643	161.9677	0.3650	0.7258	<0.0001
Albuminuria							
NLR	0.6246	0.6100	0.6392	1.3037	0.4457	0.7486	Reference
MLR	0.5835	0.5682	0.5987	0.2211	0.5105	0.6196	<0.0001
PLR	0.5470	0.5313	0.5627	112.4500	0.5046	0.5821	<0.0001
SII	0.5731	0.5578	0.5883	327.8316	0.3837	0.7396	<0.0001
SIRI	0.5897	0.5746	0.6047	0.7214	0.4749	0.6802	<0.0001
AISI	0.5454	0.5300	0.5609	162.7960	0.3585	0.7244	<0.0001
Low-eGFR							
NLR	0.6099	0.5922	0.6276	1.6172	0.5730	0.6033	Reference
MLR	0.5729	0.5544	0.5914	0.2347	0.5574	0.5591	<0.0001
PLR	0.5470	0.5286	0.5655	105.3033	0.4230	0.6638	<0.0001
SII	0.5679	0.5497	0.5862	403.1517	0.5153	0.6061	<0.0001
SIRI	0.5755	0.5574	0.5937	0.7236	0.4588	0.6667	<0.0001
AISI	0.5385	0.5200	0.5571	161.9677	0.3466	0.7253	<0.0001

^1^AUC: area under the curve.

^2^95% CI: 95% confidence interval.

## 4 Discussion

The prevalences of DKD, albuminuria, and low-eGFR with NLR were positively correlated in this cross-sectional research of 7,153 US adult T2DM patients. Subgroup analysis and interaction tests revealed that their associations were not significantly different across populations. ROC analysis showed that compared with other inflammatory markers (MLR, PLR, SII, SIRI, and AISI), NLR may have better discriminative ability and accuracy in identifying the risk of DKD, albuminuria, and low-eGFR. To sum up, we must emphasize the significance of NLR levels in evaluating kidney health in the US diabetic population.

The relationships between inflammatory markers and DKD have been investigated in a few earlier research. In a study by Wan et al., it was discovered that NLR levels were positively correlated with ACR levels and the prevalence of DKD in Chinese diabetes patients (15). The prevalence of albuminuria increased with increasing NLR levels in the Japanese diabetic population (17). NLR and PLR were revealed to be significant risk factors for predicting albuminuria in a study of diabetic individuals from Syria (24). Furthermore, the predictive usefulness of NLR for early DKD was discovered by a prospective Egyptian investigation (16). Our study has some benefits as compared to earlier research. First, we provided new evidence for the association between NLR and DKD in US diabetic patients, while previous studies mainly focused on Asian and African populations. Second, to our knowledge, no research has examined the connections between low-eGFR, DKD, and albuminuria with NLR and other inflammatory markers (MLR, PLR, SII, SIRI, and AISI) in the same diabetic population. Lastly, we used ROC analysis to evaluate the predictive power of NLR and other inflammatory markers on DKD, albuminuria, and low-eGFR. This was an essential difference between our study and previous studies.

Our research delved into the relationships between NLR and other inflammatory markers with renal function in T2DM patients. Firstly, our findings demonstrated the positive correlation between MLR and DKD. Previous studies have come to similar conclusions (25). Notably, our study introduces a novel dimension by highlighting a nonlinear link between them. When MLR < 0.43, MLR displayed a positive connection with DKD. There was no significant connection seen on the right side of the breakpoint, nevertheless. Furthermore, a 4.75-fold rise in the prevalence of albuminuria was also observed in our study for every unit increase in MLR. We also found that the prevalence of DKD increased with the SII level, in line with earlier research (26, 27). However, our investigation did not reveal a substantial connection between PLR and renal function, which differed from earlier research findings (24, 28). The reasons for these inconsistent results include differences in sample size, eGFR calculation method, population, race, and geography.

To our knowledge, no research has looked into how SIRI and AISI relate to renal function in T2DM patients. Past research primarily focused on the robust correlation between SIRI levels with CVD and peripheral arterial disease (PAD) in diabetic patients (29, 30). In our study, we demonstrated that for every one-unit increase in SIRI, the prevalence of DKD increased by 2.01-fold. According to earlier research, the increased neutrophils and monocytes and decreased lymphocytes all contribute to the onset of DKD (31–33). This might elucidate the link between SIRI and renal function. More further comprehensive prospective studies are imperative to validate and consolidate these outcomes.

The primary outcome of our investigation reveals a positive correlation between NLR and DKD in US T2DM patients. That is, the prevalence of DKD increased 1.90 times for every unit rise in NLR. Paralleling earlier research underscored the significant connection between NLR levels and albuminuria among diabetic patients (16, 17, 24). Similarly, we found that the higher the level of NLR, the higher the prevalence of albuminuria. In addition, we observed a positive and nonlinear association between NLR and low-eGFR. And a breakpoint of the threshold effect was calculated to be 1.88. When NLR > 1.88, the prevalence of low-eGFR increased 23.02 times for each unit increase in NLR. There was no proof of a meaningful relationship on the left side of the breakpoint. The previous research similarly points to the potential of NLR in forecasting deteriorating renal function in diabetic patients (34). In conclusion, the American T2DM population with higher NLR levels should be aware of kidney health. This might be because innate immunity (mediated by neutrophils) and adaptive immunity (mediated by lymphocytes) are both reflected in the easily accessible and inexpensive NLR (15). In addition, the stability of NLR is better and less affected by physiological and pathological status. The superiority of NLR has been supported by prior studies. The ROC value of NLR was significantly better than PLR in diagnosing DKD (24, 28). This conclusion was supported by our study, where ROC analysis revealed that, when compared to the other five inflammatory markers (MLR, PLR, SII, SIRI, and AISI), NLR may have the better discriminatory power and accuracy in assessing the risk of DKD, albuminuria, and low-eGFR. In conclusion, the NLR offers a great deal of potential for assessing renal function in T2DM patients in the United States as a straightforward, affordable, and frequently used inflammatory marker.

Our subgroup analysis indicated that the prevalence of DKD was significantly higher in males than in females for each unit increase in NLR. This result is in line with earlier research (35). This could be explained by either the detrimental effect of testosterone or the protective property of estrogen (36). Additionally, we discovered that the associations between NLR with DKD, albuminuria, and low-eGFR was not significantly impacted by age, sex, BMI, hypertension, or CVD. These relationships may be valid for different populations. These results confirm and add to the evidence that NLR is a risk factor threatening renal function in the community of Americans with T2DM.

Research is still ongoing to determine the possible mechanism underlying the relationship between DKD and NLR. We think this finding might have something to do with the inflammatory state associated with DKD. Research has indicated a direct relationship between systemic and renal inflammation and the pathological process of DKD. Nod-like receptor protein 3 (NLRP3) inflammasome and several inflammatory cytokines, such as interleukins and tumor necrosis factors, cause pathological changes in kidney structure through a variety of inflammatory pathways (37). They worsen renal fibrosis, tubular damage, and glomerular sclerosis in addition to raising urine albumin excretion. Traditional markers of inflammation, neutrophils are a crucial part of the innate immune response (38, 39). Renal cellular stress brought on by ongoing hyperglycemia in the early stages of diabetes triggers an innate immune response and draws leukocytes to the kidney (40). Additionally helpful in the early and advanced phases of diabetic nephropathy are macrophages and lymphocytes (41). As a result, the progression of DKD is accelerated and renal damage occurs.

Our study has several advantages. We used information from the NHANES, a comprehensive, population-based survey with stringent research procedures and quality assurance checks. The reliability and representativeness of our findings are increased by the size of our sample and the adjustment for relevant confounders. NLR is a promising tool for therapeutic usage because it is a commonly used, non-invasive, simple to use, and affordable technique. Our study does, however, have certain shortcomings. Due to the cross-sectional design of the study, we were unable to establish a causal relationship between NLR and DKD. We took a lot of significant factors into account, but it is impossible to completely rule out the impact of additional unmeasured confounders. Due to the cross-sectional survey of the US population, our results may not apply to other populations or ethnic groups.

## 5 Conclusion

Compared to other inflammatory markers (MLR, PLR, SII, SIRI, and AISI), NLR may serve as the more effective potential inflammatory marker for identifying the risk of DKD, albuminuria, and low-eGFR in US T2DM patients. T2DM patients with elevated levels of NLR, MLR, SII, and SIRI should be closely monitored for their potential risk to renal function. Nevertheless, additional thorough prospective research is required to confirm and validate these results.

## Data availability statement

The original contributions presented in the study are included in the article/supplementary material. Further inquiries can be directed to the corresponding authors.

## Ethics statement

The studies involving humans were approved by National Center for Health Statistics Research Ethics Review Board. The studies were conducted in accordance with the local legislation and institutional requirements. The participants provided their written informed consent to participate in this study. Written informed consent was obtained from the individual(s) for the publication of any potentially identifiable images or data included in this article.

## Author contributions

XL: Writing – original draft. LW: Writing – original draft, Writing – review & editing. HX: Writing – review & editing. HZ: Writing – review & editing. ML: Data curation, Supervision.
